# Core-shell gold-nickel nanostructures as highly selective and stable nonenzymatic glucose sensor for fermentation process

**DOI:** 10.1038/s41598-020-58403-x

**Published:** 2020-01-28

**Authors:** Xuejin Gao, Xinzhao Du, Danye Liu, Huihui Gao, Pu Wang, Jun Yang

**Affiliations:** 1 Faculty of Information Technology, Beijing University of Technology, Beijing, 100124 China; 2 State Key Laboratory of Multiphase Complex Systems, Institute of Process Engineering, Chinese Academy of Sciences, Beijing, 100190 China; 3 Engineering Research Centre of Digital Community, Ministry of Education, Beijing University of Technology, Beijing, 100124 China; 4 Beijing Laboratory for Urban Mass Transit, Beijing University of Technology, Beijing, 100124 China; 5 Beijing Key Laboratory of Computational Intelligence and Intelligent System, Beijing University of Technology, Beijing, 100124 China

**Keywords:** Electrochemistry, Nanoparticles

## Abstract

Non-enzymatic electrodes based on noble metals have excellent selectivity and high sensitivity in glucose detection but no such shortcomings as easy to be affected by pH, temperature, and toxic chemicals. Herein, spherical gold-nickel nanoparticles with a core-shell construction (Au@Ni) are prepared by oleylamine reduction of their metal precursors. At an appropriate Au/Ni ratio, the core-shell Au@Ni nanoparticles as a sensor for glucose detection combine the high electrocatalytic activity, good selectivity and biological compatibility of Au with the remarkable tolerance of Ni for chlorine ions (Cl^−^) and poisoning intermediates in catalytic oxidation of glucose. This electrode exhibits a low operating voltage of 0.10 V vs. SCE for glucose oxidation, leading to higher selectivity compared with other Au- and Ni-based sensors. The linear range for the glucose detection is from 0.5 mmol L^−1^ to 10 mmol L^−1^ with a rapid response time of ca. 3 s, good stability, sensitivity estimated to be 23.17 μA cm^−2^ mM^−1^, and a detection limit of 0.0157 mM. The sensor displays high anti-toxicity, and is not easily poisoned by the adsorption of Cl^−^ in solution.

## Introduction

Highly sensitive and selective detection of glucose is critical in chemical industry, clinical diagnosis, fermentation engineering and food industry, etc. During the fermentation process, glucose, as the main carbon source, plays an important role in the growth and synthesis of bacteria. In the existing glucose detection methods, except for iodometry^1^, chromatography^2^, micro-Raman spectroscopy^3^, fluorescence spectroscopy^4^, photoelectrochemical method and colorimetry^5,6^, electrochemical method has been widely used due to it can obtain detection results in real time by simple operation. Electrochemical glucose sensor accounts for approximately 85% of the biosensor industry, and is a booming realm^7^.

Conventional glucose sensors relying on immobilization of glucose oxidase (GOx) as molecular recognition elements on various substrates have been the research hotspots in the past few decades. These enzyme-based sensors usually have good selectivity and high sensitivity in glucose detection. However, the enzyme is susceptible to pH, temperature, and toxic chemicals because of the complexity of the fermentation environment^8–10^. The performance of GO_x_ sensor is also affected by the dissolved oxygen degree and the diffusion rate of hydrogen peroxide. In addition, for online glucose detection, the biosensor must be able to withstand high temperature (∼120 °C) steam sterilization in order to prevent contamination during the fermentation process. In this sense, common factors such as pH value and dissolved oxygen are often used to indirectly assess the glucose concentration.

Great efforts have been made for direct glucose determination at non-enzymatic electrodes like Pt^11–13^, Au^14–16^, transition metals and alloys^17–22^ to avoid the above-mentioned drawbacks of enzyme-based sensors. Nonenzymatic electrochemical glucose sensors based on nanosized gold have been extensively studied because of their high electrocatalytic activity, good selectivity and biological compatibility. Typically, Shu *et al*. fabricated high-quality three-dimensional (3D) Au-graphene nanocomposites through a one-step process^23^. Li *et al*. prepared macroporous Au films with higher roughness using the macroporous Cu films as templates followed by a galvanic replacement^24^. Zhou *et al*. reported a gold nanoparticle-constituted nanotube array electrode, which offers an extended linear detection range of glucose from 1 mM to 42.5 mM^25^. Nevertheless, the bare gold electrode is unable to work during long fermentation cycles due to its poisoning by chloride ions and the intermediates formed during reaction process, which block the active sites, and unable to maintain good selectivity in the fermentation environment with many impurities^26,27^. Therefore, the addition of a second metal, i.e. to generate bimetallic nanoparticles or nanocomposites^28–35^, is often used to improve the sensor performance. In principle, the second metal can effectively manipulate the electronic and geometric properties of the nanoparticles, leading to their higher selectivity and reactivity. In particular, the bimetallic nanoparticles with core-shell architectures could offer good stability and superior electronic properties, compared with other types of nanostructures. The metal shell shields the core metal from poisoning and corrosion in fermentation medium, while the strain and ligand effects of the core metal endow the shell metal with features favorable for electrocatalysis. Indeed, during the last few years, there has been a tremendous growth in the interest in applying bimetallic nanoparticles combining Cu, Ni, or Co with noble metals in catalysis^36^. Lee *et al*. introduced a macroporous Au–Pt hybrid 3D electrode fabricated by electroplating platinum nanoparticles onto the surface of the coral-like macroporous Au having a roughness factor (RF) of 2024.7^37^. Aoun *et al*. investigated the underpotential deposition of various ad-metals (Cu, Ag, Ru, Pt, Pd and Cd) on the Au electrode. They found that the Ag ad-atoms of 1/3 monolayer (ML) onto the Au(111) surface lead to a decrease in peak potential for glucose oxidation (∼0.2 V vs SCE) and its higher selectivity^38^. On the other hand, Ni electrode has also been the most widely utilized non-enzymatic electrode for determining glucose in alkaline media^39–42^. Compared to other metals, Ni-based sensors display remarkable efficiency in catalytic oxidation of glucose and are not affected by the adsorption of chlorine ions and oxidized intermediates. Xu *et al*. prepared a Ni/TiO_2_ sensor formed by a hydrothermal ion exchange method, and they found that the stability and anti-toxic properties of the prepared sensor are significantly enhanced^43^. However, the selectivity of nickel-based sensors is too low because of their catalytic capability at high operating voltage for the oxidation of various substances^44^.

In this work, taking advantages of metallic Au and Ni, we report the synthesis of spherical Au@Ni nanoparticles with a core-shell structure through a seed-mediated growth in oleylamine. In this strategy, Au nanoparticles served as seeds are prepared in advanced by oleylamine reduction of their metal precursors. Then in the presence of Au seeds, the Ni precursors are reduced for the formation of core-shell Au@Ni nanostructures. The obtained Au@Ni nanostructures supported on carbon substrates (Au@Ni/C) are characterized by XRD, XPS, TEM, and EDS. We will demonstrate that the oxidation of glucose on core-shell Au@Ni nanostructures is similar to that on the surface of pure Au particle. The core-shell nanostructures protect the active sites on the particle surface from the adsorption of chloride ions and intermediates, and the formed Ni layer allows the formation of metal-OH sites, analogous to the Au-OH sites on pure Au particle surface, at more negative potentials, which can avoid the oxidation of other interfering substances in the fermented liquid on the sensor^38^. Then the sensitivity, selectivity and stability of the prepared electrode towards glucose oxidation are evaluated through electrochemical characterizations in 0.1 M NaOH (pH = 13). The results show that the core-shell sensor may detect the glucose during fermentation process with excellent selectivity and stability, implying a great potential as an enzyme-free glucose sensor for fermentation process.

## Results and Discussion

### Characterization of the Au@Ni/C electrode

**Figure 1:**
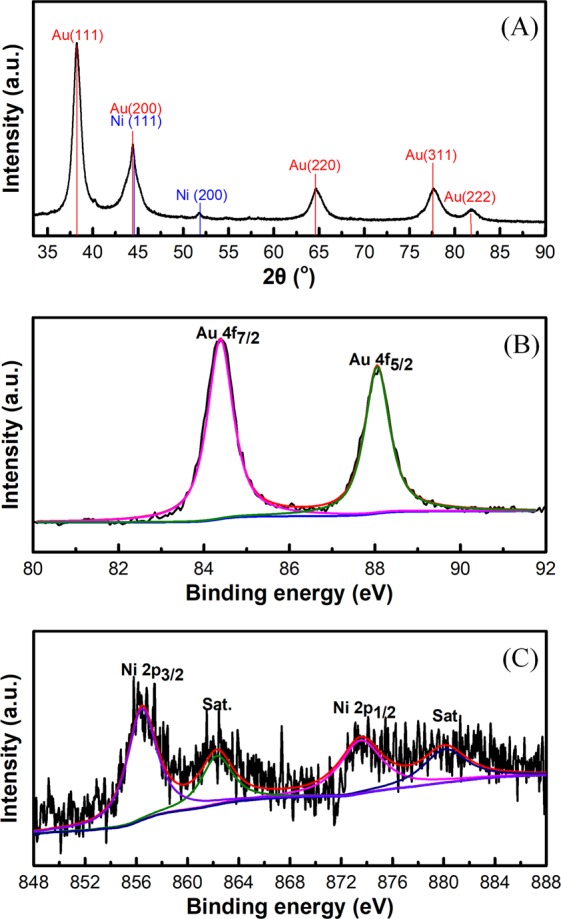
XRD pattern of Au@Ni/C electrode materials (**A**); XPS spectra of Au 4f (**B**) and Ni 2p (C) of Au@Ni/C electrode materials.

The crystalline structure and the existence of Au and Ni element in the prepared core-shell nanoparticles were investigated by XRD. Figure 1A shows two characteristic diffraction peaks at 44.5°and 51.8°, corresponding to the (111) and (200) crystalline planes of face-centered cubic (fcc) Ni phase, respectively. The other five characteristic diffraction peaks at 38.2°, 44.4°, 64.6°, 77.5°and 81.9° can be indexed to the(111), (200), (220), (311) and (222) crystalline planes of fcc Au. The XRD characterization suggests that the nanoparticles are composed of Au and Ni elements.

XPS tests of the samples were performed to investigate the composition of Au@Ni/C nanocatalyst. The XPS pattern of Au 4 f for Au@Ni-C was show in Fig. 1B, in which the two peaks center at 84.5 eV and 88.1 eV correspond to the binding energies of Au4f_7/2_ and Au4f_5/2_, respectively. The peaks centered at 857.4 eV and 873.6 eV shown in Fig. 1C correspond to the Ni metals at oxidized state, suggesting the easy oxidation of Ni in air. In addition, for Ni, the satellite peaks in its XPS spectra are also observed, as shown in Fig. 1C.

**Figure 2:**
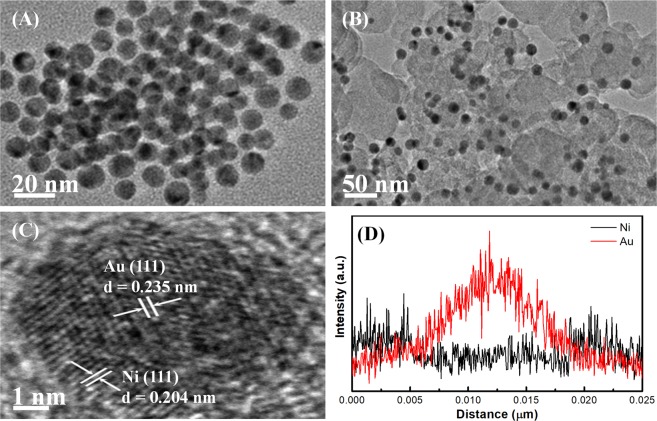
TEM images of core-shell Au@Ni nanoparticles (**A**) and Au@Ni/C samples (**B**); HRTEM image of a single Au@Ni nanoparticle on carbon substrate (**C**); the line scanning analysis of a single Au@Ni nanoparticle (**D**).

The TEM images (Fig. 2A,B) of core-shell Au@Ni nanoparticles before and after loading on carbon substrates reveal that the particles are spherical with size distribution from 7 nm to 12 nm and are evenly distributed on the carbon substrates. Figure 2C shows the high-resolution TEM (HRTEM) image, confirming that the core-shell particles have good crystallinity, in which well-defined lattice spacings of 0.235 nm and 0.203 nm at the core and shell region well match with the Au(111) and Ni(111) planes, respectively. The line scanning analysis of a single Au@Ni particle is shown in Fig. 2D, which proves that Au in the nanoparticles is mainly located in the core region, while Ni is found throughout the whole particle, definitely manifesting the formation of Au@Ni nanoparticles with a core-shell construction.

The EDS analysis was also applied to investigate the chemical composition of the as-prepared core-shell nanoparticles. Figure S1 in Supplementary Information (SI) shows the EDS spectrum of the core-shell Au@Ni particles, in which the co-presence of Au and Ni elements is clearly confirmed (among other elements appeared in the EDS spectrum, the Cu signal comes from the copper grid, while the Si and Cr are actually oxygen and a system tag error, respectively). The atomic ratios for Au@Ni/C samples prepared by growing different masses of Ni(acac)_2_ on Au seeds are 0.74/0.47 and 0.67/0.23, respectively, well consistent with the ratios in their metal precursors.

### Electrochemical measurements

**Figure 3:**
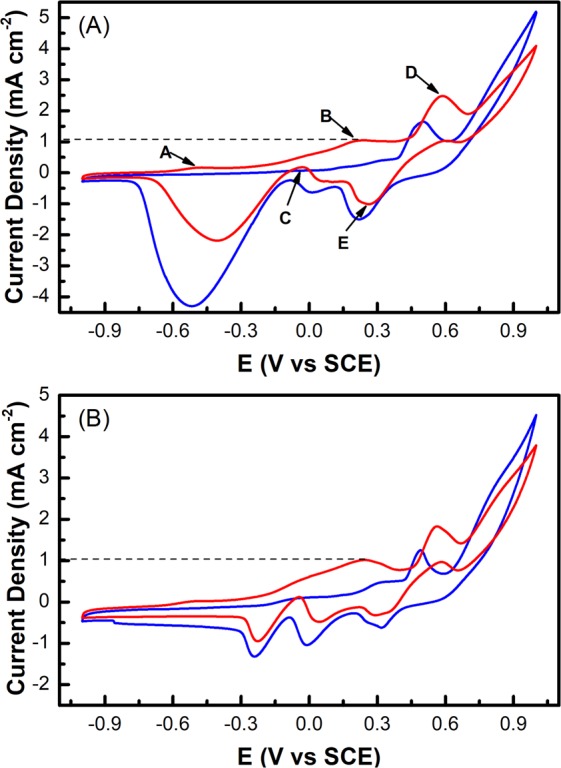
CVs of the as-prepared Au@Ni (**A**) and Au_2_@Ni electrode (**B**) in the presence (red curve) and absence (blue curve) of 10 mM glucose in 0.1 M NaOH solution with the scanning rate of 50 mV s^−1^.

The CV curves of Au@Ni/C sample were used to investigate its electrocatalytic properties towards glucose oxidation. As show in Fig. 3A, the CV curves show significant differences in current density in 0.1 M NaOH solution in the presence (red line) and absence (blue line) of 10 mM glucose. The CV curves obtained from core-shell nanoparticles show four oxidation peaks at ca. −0.5 V, −0.035 V, 0.2 V and 0.6 V and one reduction peak at ca. 0.26 V. Among them, the A, B and C peak of the CV curves indicate a typical two-step oxidation process of Au element towards glucose although a thin Ni layer has been formed on the Au surface. The electrocatalytic mechanism for the Au@Ni electrode towards glucose is a multistep one^15^. At first, the glucose molecules are dehydrogenated and adsorbed on the surface of the Au@Ni core-shell particles. Then the population of metal-OH_ads_ sites on the electrode increases with the larger potential and subsequently mediates catalytic oxidation of the intermediates toward gluconolactone.

Analogous to the glucose oxidation on pure gold surface^45^, the glucose oxidation on core-shell Au@Ni electrode highly depends on the quantity of metal-OH_ads_ and the premonolayer oxidation of metal to form metal-OH_ads_. The A peak could be attributed to the dehydrogenation of glucose to form adsorbed intermediate products. The accumulation of the intermediates due to the limited number of metal-OH_ads_ sites formed at lower potential (−0.5 V) blocks the active sites of the Au@Ni/C electrode surface, leading to the decrease of current density. The B peak at ca. 0.2 V is related to the consecutive catalytic oxidation of adsorbed intermediates because the amount of metal-OH_ads_ sites is increased. Actually, an oxidation peak should be observed at a further positive potential, corresponding to the formation of metal oxides, but it is covered by D peak. In the negative potential scan, there is an increase in current density at ca. −0.035 V because the reduction of the surface metal oxides would occur at the potential more negative than 0.2 V, and metal-OH_ads_ sites are enough for catalytic oxidation of glucose. Wang’s group has reported a similar result that there is a sharp increase in anodic current density at a potential of ca. 0.10 V^15^.

The D and E peak in CV curves associated with the conversion between Ni(II) and Ni(III), have the similar CV features for Ni-based electrode^18^. The reaction mechanism of Ni metal in alkaline medium for the electrocatalytic oxidation of glucose can be expressed as follows:

1 $${\rm{Ni}}+2{{\rm{OH}}}^{-}\to {{\rm{Ni}}({\rm{OH}})}_{2}+2{{\rm{e}}}^{-}$$

2 $${{\rm{Ni}}({\rm{OH}})}_{2}+{{\rm{OH}}}^{-}\to {\rm{NiO}}({\rm{OH}})+{{\rm{H}}}_{2}{\rm{O}}+{{\rm{e}}}^{-}$$

3 $${\rm{NiO}}({\rm{OH}})+{\rm{Glucose}}\to {{\rm{Ni}}({\rm{OH}})}_{2}+{\rm{glucolactone}}$$

As shown in SI Fig. S2A, the CVs of the Au@Ni/C electrode in 0.1 M NaOH solution containing 5 mM glucose at different scan rates were recorded to estimate the kinetics of the direct oxidation of glucose on the electrode surface. In SI Fig. S2B,C, the current densities of peak a and b are both proportional to the square root of the scan rate in the range of 10‒500 mV s^−1^, confirming that the electro-catalytic oxidation reaction of glucose is a diffusion-controlled process^15^.

By reducing the Ni ratio in core-shell Au@Ni nanoparticles (Au_2_@Ni), we further investigated the effect of Ni shell thickness on the electrocatalytic performance of the core-shell catalyst. The experiment results presented in Fig. 3B show that within the scope of the experiment, the Ni shell thickness does not significantly affect the catalytic capability, indicating within rational Au/Ni range, the core-shell Au@Ni nanoparticles as a sensor can combine the advantages of Au and Ni for glucose detection.

### Amperometric response of the Au@Ni/C to glucose oxidation

**Figure 4:**
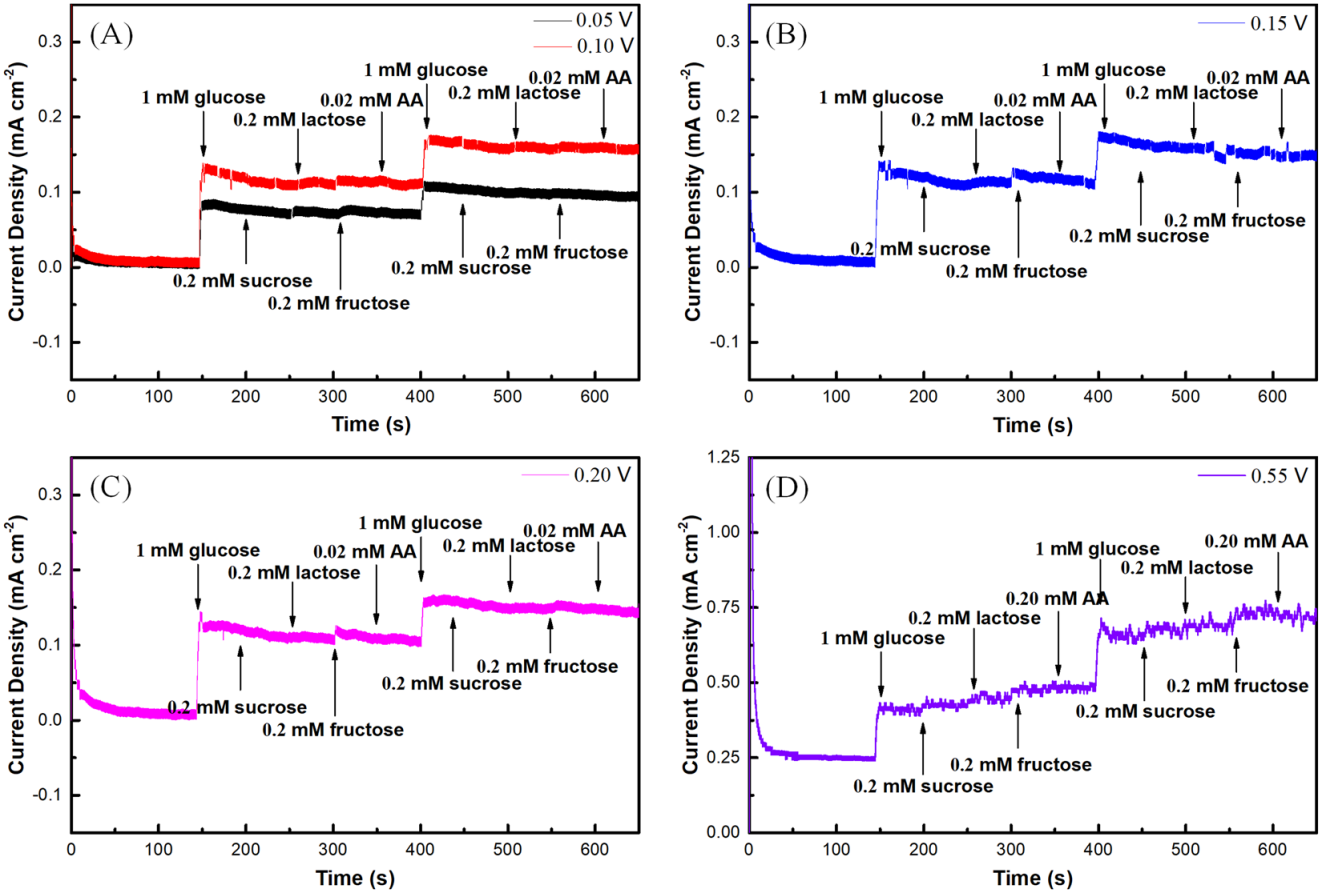
Amperometric response of an Au@Ni/C electrode to successive addition of 1 mM glucose, 0.2 mM fructose, 0.2 mM lactose, 0.2 mM and 0.02 mM ascorbic acid in a continuously stirred solution of 0.1 M NaOH at 0.05 V and 0.10 V (**A**), 0.15 V (**B**), 0.20 V (**C**), and 0.55 V (**D**), respectively.

For amperometric sensing applications, the operating voltage should be chosen to measure current response within continuous addition of glucose and the interfering species in the fermentation process at a fixed time. SI Table S1 lists composition of the substance in the fermentation broth, and the data are from Beijing Four Rings Biopharmaceutical Co., Ltd. The corn steep liquor mainly provides major elements contain C, N and trace elements such as P, Fe, K, Ca, etc. required for the growth and fermentation of culture, sometimes replaced by molasses which is also have trace substances such as vitamins, bacterial proteins and growth promoting factors. Fructose, lactose, sucrose and vitamin C (ascorbic acid, AA) in fermentation broth could be easily oxidized at a relative positive potential, and often interfere with the detection of glucose. The normal concentration of glucose is much higher than those of fructose (12~24% of molasses), lactose (11.60~19.30% of corn steep liquor, 12~24% of molasses), sucrose (24~47% of molasses) and AA (≪ 1% of molasses). Therefore, fructose, lactose, sucrose and AA are selected as the main interfering species during the experiment. The sensitivity and selectivity of the sensor was evaluated at voltage range from 0.05 V to 0.55 V with successive addition of glucose and interfering species (the ratio of glucose/fructose/lactose/sucrose/vitamin C (ascorbic acid) is 5/1/1/1/0.1) in 0.1 M NaOH soultion to determine the detection voltage. The amperometric responses of 1 mM glucose and interfering species on the Au@Ni/C electrode at different applied potentials were shown in Fig. 4. The result clearly indicates that the core-shell electrode not only has the highest response to the oxidation of glucose at 0.55 V, but also has the highest amperometric response of interfering species due to the low selectivity of nickel-based electrode for catalytic oxidation of glucose. When the potential is in the range of 0.05-0.2 V, the electrocatalytic oxidation of glucose is due to the formation of AuOH_ads_ on the Au@Ni/C electrode, which results in exaltation of the selectivity of the as-prepared electrode. As shown in Fig. 4, the addition of interferent species produces little or no significant signal compared with glucose, indicating that the Au@Ni/C electrode obtained in this study has a high selectivity for glucose sensing. Taking the sensitivity and selectivity into consideration, 0.10 V vs SCE was chosen for assessing the performance of the as-prepared sensors.

**Figure 5:**
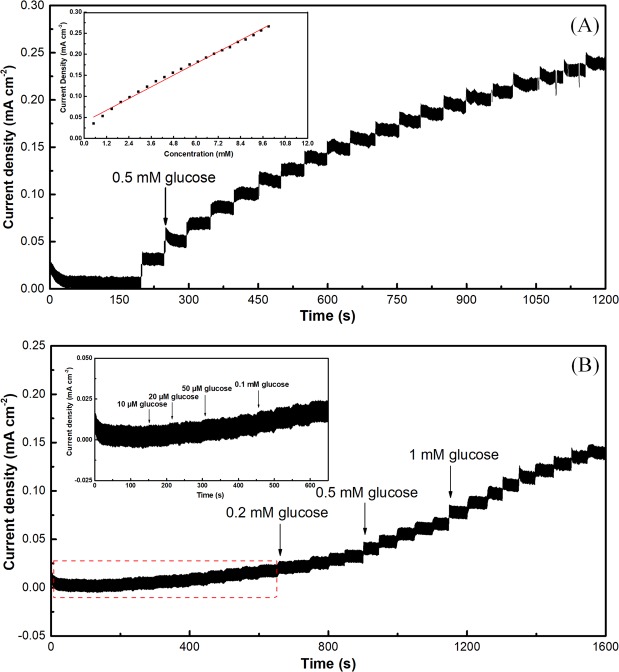
Current response of an Au@Ni/C electrode to successive addition of 0.5 mM glucose in a continuously stirred solution of 0.1 M NaOH at 0.10 V (**A**), inset shows the corresponding calibration plots; the lowest detectable concentration of an Au@Ni/C electrode towards glucose in 0.1 M NaOH solution at 0.10 V (**B**), inset is a magnified view of the curve boxed by the red frame.

Figure 5A shows the current responses of Au@Ni-C electrode to successive addition 250 μL of glucose solution (0.1 M) in 50 ml electrolyte solution at 0.10 V. The result displays a relatively wide linearity with glucose concentration ranging from 0.5 mM to 10 mM with a correlation coefficient of 0.993 and the outstanding sensitivity of 23.17 μA mM^−1^ cm^−2^. The LOD (Limit of detection) of sensors is 15.7 μM, as show in Fig. 5B and calculated by the formula in term of LOD = 3*σ*/*b*, where *σ* is the standard deviation of background signal which is obtained by measuring the current response of the Au@Ni-C sensor in the blank solution for ten times, and *b* is the sensitivity of the Au@Ni-C sensor.

The performance of Au-based and Ni-based sensors reported recently is summarized in Table 1. As compared with other sensors, The Au@Ni/C catalysts exhibit enhanced sensitivity and selectivity due to their lower operating voltage for electrocatalyzing the oxidation of glucose in comparison with other Au- and Ni-based sensors. This is due to on one hand the selective catalytic oxidation of glucose by Au, and on the other hand, the Ni ad-layer loaded onto the surface of Au NPs, whcih changes the charge of the sensor to allow the formation of more metal-OH_ads_ sites at more negative potentials^46^.

**Table 1:** Comparison of the performance of Au@Ni/C in this study and Au or Ni-based electrodes reported recently for glucose detection.

Catalysts	Applied potential (V)	Sensitivity (μA cm^−2^ mM^−1^)	Linear range (mM)	Detection limit (μM)	ref.
Au@Ni/C	0.10^a^	23.17	0.5‒10.0	15.7	This study
Au/ITO	0.15^a^	23.0	0‒11.0	5.0	^15^
Au/GtO	0.16^a^	98.7	0‒25.0	99.0	^14^
Au NPs	0.24^b^	87.5	0.1‒25.0	50.0	^47^
Au nanotube array	0.25^a^	0.0013	1.0‒42.5	10.0	^25^
Au/MWCNT/Nafion	0.30^b^	0.4	0.05‒20	20.0	^48^
Macroporous Au	0.40^b^	39.53	1‒20	25.0	^37^
Au/rGtO	0.16^a^	39.8	0‒10	63.0	^14^
Au-rGO-SWCNT	0.15‒0.25^b^	—	0‒80	0.0022	^16^
Au/NiAu MNAs	0.40^a^	483	0.005‒31	1.0	^18^
Au@Cu_2_O	0.65^a^	715	0.05‒2.0	18.0	^21^
Au-Pt hybrids	0.40^a^	39.53	1‒20	25.0	^37^
Ni-Au MCL	0.55^b^	506	0.02‒10	14.9	^49^
Au-Ni bimetal	0.40^a^	1.30	0.01‒20	0.29	^50^
Au@Pt/Au	0.35^b^	8.28	0.01‒10	0.4457	^51^

^a^The reference electrode is a saturated calomel electrode. ^b^The reference electrode is an Ag/AgCl electrode.

### Reproducibility, stability and anti-toxic of the Au@Ni/C electrode

**Figure 6:**
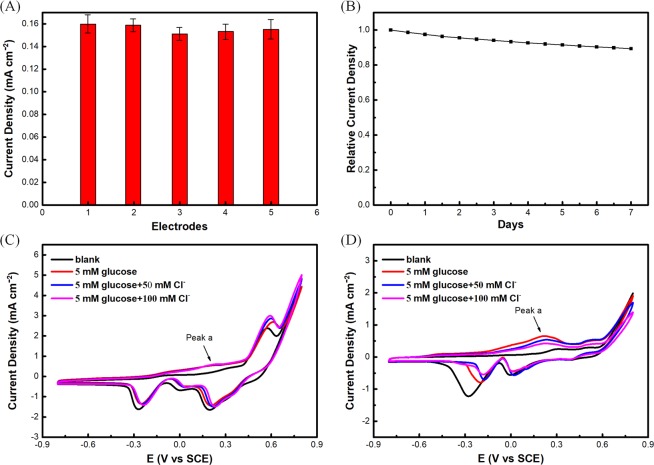
Current densities of five Au@Ni/C electrodes in 0.1 M NaOH containing 5 mM glucose at 0.10 V by amperometric measurements (The current densities were normalized to the first electrode) (**A**); current densities of 5 mM glucose in 0.1 M NaOH solution tested every half day by amperometric measurements (The current densities were normalized to the first day) (**B**), the current densities are the average values of triplicate determinations; CVs of the Au@Ni/C (**C**) and Au/C (**D**) electrode with the presence and absence of different concentration of Cl^−^ ions.

To study the reproducibility of the Au@Ni/C sensor, five electrodes prepared under the same conditions were evaluated by comparing the amperometric responses in 0.1 M NaOH solution with 5 mM glucose. Figure 6A shows that the relative standard deviation (RSD) is no more than 3.6% for the five electrodes, indicating the excellent electrode-to-electrode reproducibility.

The long-term stability of the nonenzymatic sensor is another performance indicator for a glucose biosensor. The Au@Ni/C sensor was assessed through recording the current response of 5 mM glucose at intervals over a period of one week, and the prepared electrode was stored in air when not in use. Figure 6B displays the investigation results that the measured peak current density retains more than 90% of the initial current response in continuous tests, showing that the glucose sensor has a superior long-term stability.

The anti-toxic of sensors is also a main factor that can influence the stability. The traditional gold electrodes are often adsorbed by Cl^−^, an abundant species in fermentation broth, which leads to the occupation of the active sites of Au surface and further cause inhibition of the formation of Au-OH_ads_. Figure 6C,D show the CV curves of the Au@Ni/C and Au/C electrode in the absence and presence of 50 mM and 100 mM Cl^−^ in a 0.1 M NaOH solution containing 5 mM of glucose. The results show that the presence of Cl^−^ has no significant effect on the peak current in the range from 0–0.25 V for Au@Ni/C electrode but has apparent effect on the pure Au/C electrode. The existence of Ni shell can protect the sensor from affecting by the adsorption of Cl^−^ in solution, as confirmed by the comparison of the results in Fig. 6C,D, and this is also benefit for enhancing the stability of sensor.

In this study, we have fabricated core-shell Au@Ni nanoparticles by oleylamine reduction of their metal precursors, and then constructed a non-enzymatic glucose sensor by loading these core-shell particles on carbon substrates. The Au@Ni/C sensor exhibits good anti-intereference capability to impurities in the fermentation broth, excellent stability and high tolerance to the adsorption of Cl^−^ and oxidation intermediates. Therefore, we believe it is highly applicable for glucose detection during fermentation. A real-time sampling method for practical applications is currently being investigated.

## Methods

### General materials

Tetrachoroauic acid tetrahydrate (HAuCl_4_·3H_2_O, ACS reagent, ≥47.8% Au basis) and Nickel(II) acetylacetonate (Ni(acac)_2_, 95%) were purchased from Sigma-Aldrich. Vulcan XC-72 carbon substrates were purchased from Cabot. Oleylamine (80–90%, technical grade), and Nafion solution (5% in a mixture of lower aliphatic alcohols and water) were purchased from Aladdin Reagents. D-(+)-glucose (biotech grade), D-(−)-fructose (EP grade), sucrose (molecular biology grade), α-lactose monohydrate (BC grade) and L-ascorbic acid (USP grade) were obtained from the Sangon Biotech Co. Ltd. n-hexane (99.5%), acetic acid (98%) and ethanol (99.5%) were from Beijing Chemical Works. Solutions of glucose, D-fructose, sucrose, α-Lactose monohydrate and ascorbic acid (AA) were prepared using 0.1 M NaOH solution immediately before each experiment. Additionally, all the experimental measurements were carried out at room temperature.

### Synthesis of core-shell Au@Ni nanoparticles

In a typical synthesis of core-shell Au@Ni nanoparticles, a solution of 82.37 mg (0.2 mM) of HAuCl_4_·3H_2_O in 10 mL of oleylamine was placed in a three-necked flask equipped with a condenser and heated at 110 °C in a nitrogen atmosphere under magnetic stirring for 4 h for the reduction of Au^3+^ ions by oleylamine, which also severs as the capping agent. These Au nanoparticles were then used as seeds for the formation of Au@Ni and Au_2_@Ni core-shell nanoparticles. For the synthesis of Au@Ni and Au_2_@Ni core-shell nanoparticles, different masses of Ni(acac)_2_ (51.38 mg (0.2 mM) and 25.69 mg (0.1 mM) for Au@Ni and Au_2_@Ni, respectively) were immediately added to the 10 mL of Au nanoparticle solution. The mixture was then heated to 240 °C for 1 h under the nitrogen flow with rapid magnetic stirring. After reaction, the above solution was cooled down to 160 °C and aged there for 1 h. The resulting nanoparticles were then cooled down to room temperature. The nanoparticles in the solution were purified by precipitation with ethanol, and washed twice with ethanol to remove the free ligands, and then re-dispersed in hexane.

### Fabrication of Au@Ni/C electrodes

120 mg of Vulcan XC-72 carbon substrates was added to the colloidal solution of core-shell Au@Ni nanoparticles, and the mixture was vigorously stirred for 2 h. The carbon-supported nanoparticles were precipitated by ethanol, followed by re-dispersion in acetic acid. Then the Au@Ni/C particles were placed in a three-necked flask and heated at 120 °C under rapid magnetic stirring for 2 h for removing the oleylamine on the surface of the nanoparticles. The Au@Ni/C was collected by centrifugation and washed once with ethanol and then dried at 80 °C overnight in vacuum. A glassy carbon electrode (GCE) of 5 mm in diameter was polished sequentially with slurries of 0.3 and 0.05 μm alumina, and then sonically washed sequentially in 50 wt% nitric acid, ethanol and deionized water for 1 min in each. After washing, the electrodes were dried with nitrogen gas. The prepared Au@Ni/C (14 mg) was re-dispersed in the mixed solution containing 950 μL ethanol, 50 μL deionized water and 100 μL Nafion solution (5 wt%), and then sonicated for 30 min to form a homogeneous ink. Then, 10 μL of as-prepared catalyst ink was loaded on clean GCE followed by drying in air.

### Sample characterizations

Transmission electron microscopy (TEM) and high resolution TEM (HRTEM) were performed on the JEOL JEM-2100F electron microscope. A drop of the nanoparticle solution was dispensed onto a 3 mm carbon-coated copper grid for the TEM measurements. Energy dispersive spectrometer (EDS) analysis was used to analyze the chemical compositions of the synthesized nanoparticles. Powder X-ray diffraction (XRD) patterns were recorded on a Rigaku D/Max-3B diffractometer with Cu K alpha radiation (λ = 0.15406 nm). X-ray photoelectron spectroscopy (XPS) analyses were conducted on a VG ESCALAB MKII spectrometer.

### Electrochemical measurements

The Electrochemical measurements were carried out in a lab-made electrochemical cell at room temperature, connected to a Bio-logic VMP1 potentiostat. The saturated calomel electrode (SCE) and platinum mesh electrode (1 × 1 cm^−2^) were used as the reference electrode and counter electrode, respectively. Cyclic Voltammogram (CV) performed in the potential range from −1.0 V to 1.0 V vs. SCE with a scan rate of 50 mV s^−1^ was used to observe the onset-potential of prepared sensor towards the catalytic oxidation of glucose to gluconolactone. The amperometric technique was carried out in the applied potentials chosen from the CV results for assessing the performance of Au@Ni/C sample towards glucose oxidation. The current obtained in the above experiment was recorded when the transient reaches a steady state.

## Supplementary information

SREP-19-39398-SI.
