# Supplementary material for: Core-shell gold-nickel nanostructures as highly selective and stable nonenzymatic glucose sensor for fermentation process
Source: Sci Rep. 2020 Jan 28;10:1365. doi: 10.1038/s41598-020-58403-x (PMC6987199; doi:10.1038/s41598-020-58403-x)
Supplement: Supplementary file 1 — SREP-19-39398-SI. [file 41598_2020_58403_MOESM1_ESM.doc]

Supplementary Information

**Core-shell gold-nickel nanostructures as highly selective and stable nonenzymatic glucose sensor for fermentation process**

Xuejin Gao^1^, Xinzhao Du^1^, Danye Liu^2^, Huihui Gao^1,3,4,5^, Pu Wang^1,3,4,5^, and Jun Yang^2^,*

^1^Faculty of Information Technology, Beijing University of Technology, Beijing, 100124, China

^2^State Key Laboratory of Multiphase Complex Systems, Institute of Process Engineering, Chinese Academy of Sciences, Beijing 100190, China. Fax: 86-10-8254 4915; Tel: +86-10-8254 4915; E-mail: [jyang@ipe.ac.cn](mailto:jyang@ipe.ac.cn)

^3^Engineering Research Centre of Digital Community, Ministry of Education, Beijing University of Technology, Beijing, 100124, China

^4^Beijing Laboratory for Urban Mass Transit, Beijing University of Technology, Beijing, 100124, China

^5^Beijing Key Laboratory of Computational Intelligence and Intelligent System, Beijing University of Technology, Beijing, 100124, China

**Table S1.** The composition of a common fermentation medium.

| Medium | Seed medium | | Fermentation medium | |
| --- | --- | --- | --- | --- |
|  | Substance | Concentration (g L^‒1^) | Substance | Concentration (g L^‒1^) |
|  | Starch | 30.0 | Glucose | 10.0 |
|  | Glucose | 10.0 | Corn steep liquor | 40.0 |
|  | Peptone | 4.0 | (NH_4_)_2_SO_4_ | 5.67 |
|  | Corn steep liquor | 2.0 | K_2_HPO_4_ | 4.0 |
|  | K_2_HPO_4_ | 0.5 | MgSO_4_⋅7H_2_O | 35.0 |
|  | MgSO_4_⋅7H_2_O | 0.5 | KH_2_PO_4_ | 4.53 |
|  | NaCl | 0.5 |  |  |
|  | Distilled water | 1000 mL L^‒1^ |  |  |

**Figure S1.** The EDS spectrum of core-shell Au@Ni nnaoparticles on carbon substrates.

**Figure S2.** Scan rate dependence of CV curves for an Au@Ni/C electrode in a solution of 0.1 M NaOH + 5 mM glucose (A). Scan rate: 10, 20, 50, 80, 100, 150, 250, 500 mV s^‒1^; the dependence of peak current of peak a (B) and peak b (C) on the square of scan rate.
